# The mediating role of psychological capital on the relation between distress and empathy of medical residents: a cross-sectional survey

**DOI:** 10.1080/10872981.2019.1710326

**Published:** 2020-01-03

**Authors:** Jing Jin, Honghe Li, Wenwen Song, Nan Jiang, Weiyue Zhao, Deliang Wen

**Affiliations:** Institute for International Healthcare Professionals Education and Research, China Medical University, Shenyang, China

**Keywords:** Medical residents, empathy, distress, psychological capital, mediating role

## Abstract

**Background**: Medical residents usually suffer from work overload and experience both personal and professional distress, which affects their level of the empathy to patients. Psychological capital (PsyCap) is a psychological resource that is negatively associated with indicators of distress.

**Objective**: This study explored the potential mediating effect of PsyCap on the relationship between distress and empathy, which may help healthcare professionals in their defense of empathy erosion due to distress.

**Design**: A total of 620 first-year residents were recruited for this cross-sectional survey. Empathy and PsyCap of residents were assessed by the Chinese version of the Jefferson Scale of Physician Empathy and the Psychological Capital Questionnaire, respectively. In this study, both personal and professional aspects contributing to resident distress were investigated by the Satisfaction with Life Scale and an occupational distress scale. T-tests and one-way ANOVA were used to test differences in empathy of residents. Pearson’s correlation was used to examine correlations between distress, PsyCap, and empathy. Structured equation modeling was used to conduct the pathway analysis to test the mediating effect of PsyCap on the association between distress and empathy.

**Results**: 537 residents (86.6%) completed the survey. Distress, empathy, and PsyCap were significantly correlated (*P* < .01) and in the expected directions. The first step analysis showed that as distress increased, the empathy of residents significantly decreased (*P* < .01), with the direct effect coefficient being 0.265. When PsyCap was included, the direct effect coefficient decreased to 0.033. This indirect effect was significant (*P* < .01). The variance accounted for was 81.14%, which indicated a partial mediating effect of PsyCap.

**Conclusions**: PsyCap may serve a significant protective role against the impact of distress on the level of empathy of medical residents. In addition to reducing distress, PsyCap development could be considered in empathy decline prevention and empathy cultivating strategies.

**Abbreviations:** PsyCap: Psychological capital; JSPE: Jefferson Scale of Physician Empathy; PCQ: Psychological Capital Questionnaire; SWLS: Satisfaction with Life Scale; VAF: Variance accounted for; SD: Standard deviation.

## Introduction

Empathy of medical professionals has been defined by Hojat et al as ‘a predominantly cognitive attribute that includes an understanding of experiences, concerns, and perspectives of the patient’ [1,2]. Empathy has been a particularly therapeutic element of patient-physician communication, which can increase diagnostic accuracy [3,4], decrease patient complaints and grievances [5,6], and improve patient quality of life [7]. However, a large number of longitudinal and cross-sectional studies have demonstrated that there were significant declines in empathy as time in medical practice progressed [8]. Residency training is a key transition period from medical student to physician, during which the resident will face unprecedented emotional, mental, and physical challenges. Especially for junior residents, the initial adaptation period and high workload could exacerbate this stress. Due to the rigorous activities in clinical practice, distress has been identified as a main influencing factor of empathy decline by many studies in medical students and residents [9–12]. Some experts have postulated that distress impacts medical professionals’ empathy by producing a psychological response that focuses on survival and self-protection as a coping mechanism [13,14]. With the extended focus on self during times of distress, it would be much more difficult for residents to be understanding of and attentive to patients’ emotional needs.

Distress is a psychological state in which a person is unable to completely adapt to stress and shows maladaptive behaviors [14]. For medical residents, distress is usually caused by the stress of medical liability, imbalance between personal and professional lives, and even potential negative effects of the hidden curriculum [15]. Distress (e.g. depression, burnout, reduced quality of life) in medical professionals can ultimately damage their level of empathy with patients [6]. Moreover, the distress caused by inevitable events such as the anxiety of first contact with patient, patient death, stress of role modeling, and work overload calls for strong mental and emotional capacities from medical students and residents. This necessitates research exploring psychological factors that may alleviate the negative effects of distress on empathy in health professionals and medical students.

Psychological capital (PsyCap) is ‘the positive psychological state of a person in the process of growth and development, which goes beyond human capital and social capital, and is a psychological resource to promote personal growth and performance improvement’, defined by Lythans [16]. It includes four domains: self-efficacy, optimism, hope, and resiliency [17], which are embodied in individual growth and development on a daily basis. Working professionals who have high PsyCap apply themselves to better handle challenges, are more optimistic in negative situations, expect positive outcomes, and quickly recover after setbacks. Previous studies have shown that PsyCap is positively correlated with work performance, job satisfaction, and level of well-being [17,18] and is negatively associated with depression, anxiety, burnout, and pressure [18–21], which are known factors of distress in medical professionals [22–25]. PsyCap has also been considered to be a positive resource in combating destructive emotions, pressure, burnout, and work-life conflicts [17,21,26]. This calls for research on PsyCap of medical residents or students from different countries and healthcare systems, Two 2018 studies have already investigated the PsyCap of Chinese medical residents and indicated that PsyCap has a significant negative relationship with work stress or perceived stress [27,28]. Moreover, the mediating role of PsyCap on occupational stress, burnout, and work-life balance has been demonstrated by a number of studies in China [19,29,30]. Therefore, a high PsyCap may help reduce the inevitable distress in residents and thereby allow for improved empathy and physician-patient communication.

Despite these current findings, PsyCap has yet to be included in the medical curriculum as an element of medical training or remediation. Thus, this study aims to explore the relationship between PsyCap and empathy and to evaluate the moderating effect of PsyCap on the relationship between distress and empathy. The conceptual framework was constructed based on the hypothesis presented above regarding distress, PsyCap, and empathy. Directional hypotheses for this framework are as follows: H1: as distress increases, empathy of residents is expected to decrease; H2: as PsyCap increases, distress of residents is expected to decrease; H3: after controlling for distress as the predictor variable, empathy of residents is expected to increase as PsyCap increases; H1ʹ: associations between distress and empathy after adding PsyCap as a mediator. This framework suggests that PsyCap may have a mediating effect on the relationship between distress and empathy and that higher PsyCap scores may mitigate the negative impact of distress on resident empathy. Sufficient evidence of the PsyCap effect on the relationship between distress and empathy will provide a good reference for medical educators or policy makers to add PsyCap element to the medical professionalism curriculum and remediation process.

## Materials and methods

### Study sample and procedure

A total of 620 first-year residents from Dalian Medical University were recruited for this cross-sectional survey in September 2016. The current Chinese clinical medical education system comprises five years undergraduate education and three years compulsory residency training. Five trained investigators conducted the survey and explained the purpose of the study and the guidelines for filling out the questionnaires to the participants. The four part questionnaire was self-administered, and demographic information, including sex, age, and field of study, was collected on administration. Participants had the right to deny the survey and to withdraw from the study at any time.

### Questionnaire

#### The Jefferson scale of physician empathy (JSPE)

The JSPE was developed by Hojat *et al* as a self-reporting scale to assess empathy and had been used in many countries [31–33]. This study used the Chinese version of the JSPE [34]. The JSPE contains 20 items with a 7-point Likert scale for each item. The total score was obtained as a sum of all item scores, with values ranging between 20 and 140 and higher values indicating a higher level of empathy.

#### The psychological capital questionnaire (PCQ)

This study used the Chinese version of the PCQ [17] to assess PsyCap. It includes 24 items, and the total scores for the PCQ ranged between 24 and 144, with higher scores meaning a higher level of PsyCap. The PCQ contains four dimensions: self-efficacy, hope, resilience, and optimism. There are six items in each of the four dimensions, which were answered on a 6-point Likert scale from strongly disagree to strongly agree. The PCQ had been used in and showed acceptable reliability and validity in various populations [17,19,35,36]

#### Self-reported distress

In a systematic review of empathy, Neumann *et al* suggested that distress, in light of factors of empathy, usually consisted of burnout, lower quality of life, and depression [6]. In this study, both personal and professional aspects contributing to resident distress were investigated. Personal distress was assessed by the Satisfaction with Life Scale (SWLS) developed by Vassar et al. [37], and an occupational distress scale was developed for this study to measure work-related distress. The total score consisted of both parts and ranged between 9 and 56, with higher values indicating a lower level of self-reported distress. Detailed descriptions of the scales measuring self-reported distress are as follows:

#### Satisfaction with life scale (SWLS)

The SWLS is a 5-item instrument for self-administered assessment of subjective personal well-being. The questions were answered on a 7-point Likert scale (from 1 = strongly disagree to 7 = strongly agree), with the total score ranging between 5 and 35. Investigations in various populations provided satisfactory validity evidence for the SWLS and indicated the feasibility for its use [37–39]. The Chinese version, which was used in this study, has been demonstrated to be reliable in the Chinese population [40].

#### Occupational distress scale

Occupational distress investigated in many studies usually includes level of burnout, work-life imbalance, and required work effort. Most of these studies used lengthy instruments, sometimes even needing multiple tools to assess the concept of occupational distress [41–43]. To balance the number of items investigating occupational distress with those investigating personal distress in this study, only single items were used to reflect each domain of occupational distress as a whole, such as burnout, stress, career satisfaction, work-life balance. Thus, a concise occupational distress scale was developed for this study to assess workplace related distress and consisted of the following 4 items: (1) Satisfaction with career, ranging from 1 (strongly dissatisfactory) to 4 (strongly satisfactory). We used an even number of options for this item to eliminate the tendency to select a neutral response; (2) Frequency of burnouts with the job, ranging from 1 (almost every day) to 7 (Never). This item comes from the single question with the highest factor loading on the Maslach Burnout Inventory [44]; (3) Frequency of work-related pressure, ranging from 1 (almost every day) to 5 (less than once in the past three months); (4) Balance between job and life, ranging from 1 (very hard to balance) to 5 (very easy to balance). The total score for occupational distress ranged between 4 and 21, with higher values indicating a lower level of occupational distress (Additional file 1).

Cronbach’s α for the overall distress scale in this study was assessed and determined to be 0.771, which is an acceptable internal consistency for the questionnaire. The Kaiser–Meyer-Olkin (KMO) analysis yielded an index of 0.799. The result for Bartlett’s test of sphericity was 1309.308, which was highly significant (*P* < 0.01). Two factors emerged by Exploratory Factor Analysis and accounted for a total of 53.61% of the variance. Factor 1 was based on SWLS’s five items, with factor coefficients greater than 0.50 and accounting for 31.48% of the variance. Factor 2 was the four items from the occupational distress questionnaire, which accounted for 22.13% of the variance. Thus, with regards to validity evidence from the internal consistency test and from exploratory factor analysis, the distress scale was considered to fit the purpose of the questionnaire design and to be suitable to assess residents’ level of distress.

### Statistical analysis

There were only 23 questionnaires with missing data, only accounting less than 5% of the total response. Thus, the questionnaires with missing data were excluded from the analysis in this study. T-tests and one-way ANOVA were used to test differences in empathy of residents among categorical groups. Pearson’s correlation was used to examine correlations among distress, PsyCap, and empathy. Based on Baron and Kenny’s procedures for mediational hypotheses, mediating effects of PsyCap can be explored when Pearson correlations of the variables were significant and in the hypothesized directions [45]. All above analyses were conducted using SPSS 21.0 for Windows, and statistical significance was defined as *P* < .05. Pathway analysis was used to explore the mediating effects of PsyCap on the relationship between self-reported distress and empathy of residents. To avoid multicollinearity, all study variables were centralized before being added into the pathway analysis model [45,46]. Structured Equation Modeling on AMOS 21.0 was used to conduct the pathway analysis to test the direct effect of distress on empathy, the indirect effect of distress on empathy via PsyCap, and the total effect of distress on empathy with PsyCap included. In the first step, the focus was on the direct relationship between distress and empathy. Subsequently, the mediator (PsyCap) was added, and the full structural model was analyzed.

### Ethical approval

This study was approved by the Bioethics Academic Commission of China Medical University, Shenyang, China. Participants signed written informed consents before partaking in the investigation. According to the terms of the written informed consent, participants’ personal information were kept confidential, which were only used for the research purposes and not made available publicly. The survey was self-administered. All participation was voluntary and anonymous, and participants were not compensated.

## Results

### Description of the characteristics of the sample

Among the 620 residents, 537 returned completed surveys, which was an overall effective response rate of 86.6%. The basic characteristics of the residents (sex and specialization), along with their respective distress, PsyCap, and empathy scores, are shown in Table 1. Participant ages ranged from 21 to 33 (mean = 24.4, SD = 1.5). On average, male residents (mean = 107.10, SE = 1.06) reported statistically significant higher PsyCap level than female residents (mean = 104.49, SE = 0.75) (*P* < .01,). The Cohen’s effect size estimate was 0.25. Distress, PsyCap, and empathy had no statistically significant correlation with, sex, age, or specialization (*P* > .05).

**Table 1.** Demographic characteristics and distress, PsyCap, and empathy scores of residents

Variables	N	%	Distress (Mean±SD)	PsyCap (Mean±SD)	Empathy (Mean±SD)
**Sex**					
Male	187	33.69%	31.92 ± 7.83	107.10 ± 14.32*	106.69 ± 12.42
Female	332	59.82%	33.30 ± 7.67	104.49 ± 13.60	107.28 ± 11.13
**Specialization**					
Internal Medicine	185	33.33%	32.03 ± 7.08	103.39 ± 12.16	106.36 ± 11.42
Surgery	128	23.06%	31.81 ± 8.24	108.81 ± 12.63	106.14 ± 11.67
Obstetrics and Gynecology	30	5.40%	34.83 ± 10.16	103.83 ± 17.01	109.00 ± 11.86
Pediatrics	16	2.88%	33.00 ± 7.34	98.06 ± 10.20	108.31 ± 11.85
Dentistry	48	8.65%	35.01 ± 6.81	107.00 ± 17.75	110.17 ± 10.94
Other^✝^	147	26.49%	32.57 ± 7.74	105.82 ± 14.26	107.18 ± 11.94

*P < 0.01 (two-tails).

^✝^Others include: Medical Imaging, Medical Laboratory Science, etc.

### Correlations among distress, PsyCap, and empathy

The mean and standard deviations of distress, empathy, and PsyCap and their Pearson correlation coefficients are presented in Table 2.

**Table 2.** Means, standard deviations, and Pearson correlations of variables

Variable	Mean	SD	Empathy	Distress	PsyCap
Empathy	107.06	11.61	1		
Distress	32.57	7.75	0.17*	1	
PsyCap	105.61	13.92	0.34*	0.429*	1

*P < 0.01 (two-tails).

Results revealed statistically significant correlations among distress, empathy, and PsyCap in the expected directions (*P* < .01), which met the conditions for Baron and Kenny’s procedures for mediation [37]. However, the coefficient of determination of correlation between distress and empathy is less than 0.1.

### The mediating effect of PsyCap in the association between distress and empathy

First step analysis showed that lower levels of distress had a significantly positive effect on empathy (*P* < .01), with the direct effect coefficient being 0.265. When PsyCap was included in the full structural model, the direct effect of the relationship between distress and empathy decreased from 0.265 to 0.033. Table 3 presents results of the mediating effect. The indirect effect was significant (*P* < .01). The variance accounted for (VAF) was 81.14%, which indicated that there was a partial mediating effect [47].

**Table 3.** Analysis of the mediating effect of PsyCap between distress and empathy

Relation	Direct effect	Indirect effect	Total effect	VAF
Distress to Empathy	0.265			
Distress to Empathy including PsyCap	0.033	0.142	0.175	81.14%

## Discussion

Medical residency training is a key transformation period from medical student to practicing physician. Residents usually suffer from work overload and experience both personal and professional distress, which may damage their level of the empathy towards patients [48,49]. In this study, we hypothesized that PsyCap may be a psychological resource that can help residents defend against empathy erosion. Thus, we investigated the PsyCap of medical residents and its relationship with distress and empathy. There was a statistically significant indirect effect between distress and empathy when PsyCap was added as the mediating variable, where the direct effect of the relationship between distress and empathy decreased, indicating a partial mediating effect of PsyCap. The PsyCaps of male residents were significantly higher than the PsyCaps of female residents. Further understanding of the significance of PsyCap in residents and the relationships between distress and empathy and between distress and PsyCap could help to explain the mediating role of PsyCap on distress and empathy.

This study is the first of its kind to provide information on the PsyCap of medical residents (mean = 105.61, SD = 13.92). Compared to that of Chinese nursing students, university teachers, and physicians, residents have a higher mean PsyCap score [19,35,36]. Male residents had a higher mean PsyCap score (*P* < .05). Women may be more vulnerable to the negative routinization of the work environment than men [50]. These include but are not limited to factors such as occupational self-direction, job demands, and social support for distress [50]. Besides, one study in China also indicated that various components of PsyCap were found to play different roles in mediating the relationships between gender role orientation and job and career satisfaction [51]. Therefore, gender differences have significant influence on PsyCap and should be taken into consideration when exploring PsyCap development strategies and practices.

This study is also the first to offer empirical evidence on the impact of distress on empathy among the medical resident population. The correlation yielded a statistically significant *p* value of less than .05, which suggested that residents’ distress may exert a negative influence on empathy and hinder their engagement with patients. However, it is important to note that correlation values and coefficients of determination do not necessarily imply a significantly meaningful relationship between distress and empathy of residents. Correlation analyses from previous studies reported that the relationship between distress and empathy among medical students and physicians were statistically significant [11,52], which are consistent with the results in this study. Due to the lack of reporting of effect size in these previous studies, we cannot know how significant these correlations really are. On the correlation of sub-scale values of distress and empathy, multiple studies reported only parts of the sub-scale, such as burnout or depression, which either showed statistically significant correlations with each other or with empathy [11,53,54]. This can help explain the small effect size of correlations in this study. Distress became a more generalized concept to evaluate when occupational and personal factors were combined. Some components may play a significant role in this relationship, and some may not. In future studies, we hope to do further research on extracting and assessing specific components of distress in order to identify the key markers that play a significant role on empathy.

Results from both correlation analysis and pathway analysis indicated that PsyCap had a negative correlation with distress, which provided evidence to accept hypothesis H2 (as PsyCap increases, distress of residents is expected to decrease). This relationship between distress and PsyCap was consistent with studies that illustrated the positive influence of PsyCap on distress in Chinese nurses and people living with HIV/AIDS who were employed full-time [55,56]. With multiple studies concluding that PsyCap is negatively correlated with distress, improving PsyCap of medical residents may act as a protective factor against the varying levels of distress that are experienced during residency. Further investigation on the protective factor of PsyCap at a global level is warranted.

The indirect effect contributed by PsyCap accounted for 81.14% of the total effect of the association between distress and empathy, which indicated that PsyCap had a partial mediating role in the association between distress and empathy in medical residents. In previous studies, PsyCap played a mediating role in multiple associations between psychological variables, exhibiting a protective effect against negative psychological factors, such as stress and negative life events [19,35,36]. Although the results of this study showed only a partial mediating role of PsyCap, it still accounted for over 80% of the total effect, and the inclusion of PsyCap as a mediating variable decreased the direct effect of distress on empathy to a mere 0.033. This showed the significance of PsyCap in maintaining levels of empathy in the medical profession, especially in that of medical residents. To develop effective medical education and training programs, PsyCap development would serve as a key factor that would act as a defense against the distress experienced by medical residents. It is possible that if the PsyCap score reached a certain threshold, distress could then have only a minimal or even non-significant impact on empathy. Further confirmative studies on the optimum PsyCap score in this respect would be needed.

This study has two limitations, which have to be acknowledged. Firstly, we did not follow a rigorous systematic method in developing the occupational distress questionnaire in this study. However, psychometric properties of the occupational distress questionnaire were at generally acceptable levels. This was because items assessing occupational distress were derived from various existing occupational distress instruments that already have validity evidence [41–44]. Since these instruments were either too lengthy or too in depth, a concise version was adapted for this questionnaire to balance the distribution of empathy, PsyCap, and distress items. Secondly, the study was a cross-sectional study, which has a limited capacity to assess the causal relationships among different study variables.

## Conclusions

PsyCap acts as a protective factor against the negative effect of distress on the level of empathy in medical residents. PsyCap scores in male residents were also significantly higher than that of female residents. Prospective studies on the optimum level of PsyCap needed to be attained by medical students prior to entering residency would prepare future medical residents for the amount of personal and professional distress they face during their residency training.

## Data Availability

The datasets generated and/or analyzed during the current study are not publicly available due to concerns about the potential ability to identify individual participants using their demographic information and survey answers but are available from the corresponding author on reasonable request.
